# Supplementary material for: OpenCell: A low-cost, open-source, 3-in-1 device for DNA extraction
Source: PLoS One. 2024 May 2;19(5):e0298857. doi: 10.1371/journal.pone.0298857 (PMC11065270; doi:10.1371/journal.pone.0298857)
Supplement: S1 Table — The appropriate slicer settings for each respective part. (PDF) [file pone.0298857.s005.pdf]

Supporting Information: S1 Table - OpenCell: A Low-cost, Open-Source, 3-in-1 device for DNA Extraction

Aryan Gupta[1], Justin Yu[2], Elio J. Challita[3,4], Janet Standeven[3,5] M. Saad Bhamla[3*]

1 School of Electrical & Computer Engineering, Georgia Institute of Technology, 777 Atlantic Drive NW, Atlanta, GA, 30332, USA
2 School of Biological Sciences, Georgia Institute of Technology, 310 Ferst Dr NW, Atlanta, GA 30332, USA
3 School of Chemical & Biomolecular Engineering, Georgia Institute of Technology, 311 Ferst Drive NW, Atlanta, GA, 30332, USA
4 George W. Woodruff School of Mechanical Engineering, Georgia Institute of Technology, 801 Ferst Drive NW, Atlanta, GA, 30318, USA
5 Lambert High School, Suwanee, Georgia, United States of America

S1 Table

Component Files and Slicer Settings

STL Component Files

The following github link contains all necessary STL files for OpenCell: Github Repo

3D Printing Defects

Defects and print defects are a common occurrence in 3D printed components and can substantially impact the strength and performance of printed components. Here are some useful external resource for identifying and correcting for common defects: Simplify3d.com, all3dp.com

Slicer Settings

Component	Layer Height	Perimeters/ Walls	Support	Infill	Special
**Face**	0.24	3	**no**	20%	
**Lid**	0.24	5	**no**	30%	
**Chamber**	0.24	5	**yes-everywhere**	30%	
**Electronics Lid**	0.24	3	**no**	20%	
**Upper Arm**	0.2	3	**yes - touching buildplate**	20%	
**Lower Arm**	0.2	3	**no**	20%	
**Tertiary Gear**	0.2	5	**no**	100%	**Rectilinear Infill**
**Secondary Gear**	0.2	5	**no**	20%	
**Motor Mount**	0.2	3	**no**	30%	
**Tube Holder**	0.16	3	**no**	20%	
**Tube Lid**	0.16	3	**no**	20%	
**Centrifuge**	0.24	3	**yes - touching buildplate**	20%	
**Button Holders**	0.24	3	**no**	20%	

Detailed Component Cost Table for OpenCell

Component	Description	Price	Retail Link
Arduino Nano	Compact microcontroller	$4.25	[Link]
Brushless Motor	2204 to 2207 2300kv+ Drone Motor	$8.00	[Link]
ESC	Electronic Speed Controller	$10.00	[Link]
Display	i2C LCD Display	$2.69	[Link]
Buttons	2x Inputs to control OpenCell	$0.21	[Link]
Potentiometer	Input Device to control OpenCell	$0.50	[Link]
Hall Effect Sensor	2x Magnetic Field Detector	$0.70	[Link]
Small Bearings	4x 693ZZ bearings	$1.90	[Link]
Large Bearings	2x 608ZZ bearings	$0.85	[Link]
Breadboard	Base for the circuits	$1.16	[Link]
Wires	Used to make electronic connections	$0.75	[Link]
Magnets	14 6x3mm magnets	$0.76	[Link]
Fasteners	4, 8, 10, 12, and 16mm M3 bolts and nuts	$0.35	[Link]
Master Switch	Inline xt60 switch	$2.99	[Link]
DC Power Supply	6-12v, 2amp DC Power Supply	$9.99	[Link]
XT60 to JST Adapter	Power pass-through for Arduino	$1.97	[Link]
LiPo Battery (Optional)	3s 11.1V 2200mAh For battery operation	$11.50	[Link]
Filament Cost	Total 425g PLA Filament Cost	$4.25	
**Total**	Excluding batteries	**$49.35**	

This table provides a detailed breakdown of the cost of each component used in the OpenCell platform. The prices reflect the market costs in 2022. The total cost of the OpenCell platform, excluding the optional LiPo battery, is approximately $47. With the optional LiPo battery and excluding the DC power supply, the total cost is less than $50.
