# Supplementary material for: OpenCell: A low-cost, open-source, 3-in-1 device for DNA extraction
Source: PLoS One. 2024 May 2;19(5):e0298857. doi: 10.1371/journal.pone.0298857 (PMC11065270; doi:10.1371/journal.pone.0298857)

Supporting Information: S1 Figure - OpenCell: A Low-cost, Open-Source, 3-in-1 device for DNA Extraction

Aryan Gupta[1], Justin Yu[2], Elio J. Challita[3,4], Janet Standeven[3,5] M. Saad Bhamla[3*]

1 School of Electrical & Computer Engineering, Georgia Institute of Technology, 777 Atlantic Drive NW, Atlanta, GA, 30332, USA
2 School of Biological Sciences, Georgia Institute of Technology, 310 Ferst Dr NW, Atlanta, GA 30332, USA
3 School of Chemical & Biomolecular Engineering, Georgia Institute of Technology, 311 Ferst Drive NW, Atlanta, GA, 30332, USA
4 George W. Woodruff School of Mechanical Engineering, Georgia Institute of Technology, 801 Ferst Drive NW, Atlanta, GA, 30318, USA
5 Lambert High School, Suwanee, Georgia, United States of America

*saadb@chbe.gatech.edu

Contents

S1 Figure: Wiring diagram **2**

S2 Figure: PID Accuracy **3**

S1 Figure

Wiring Diagram of all electronic components used in OpenCell

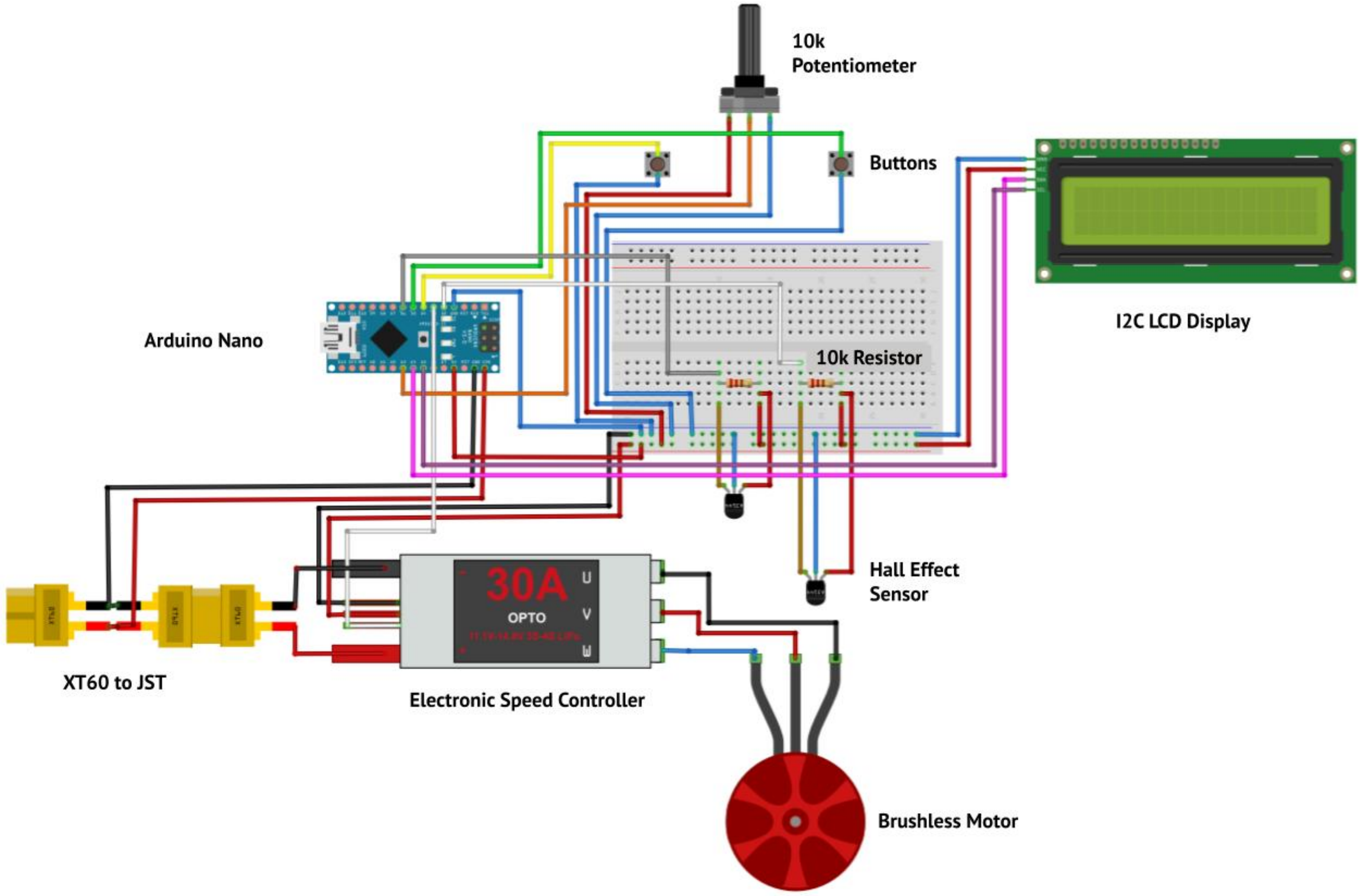

S2 Figure

Accuracy of PID controller over time

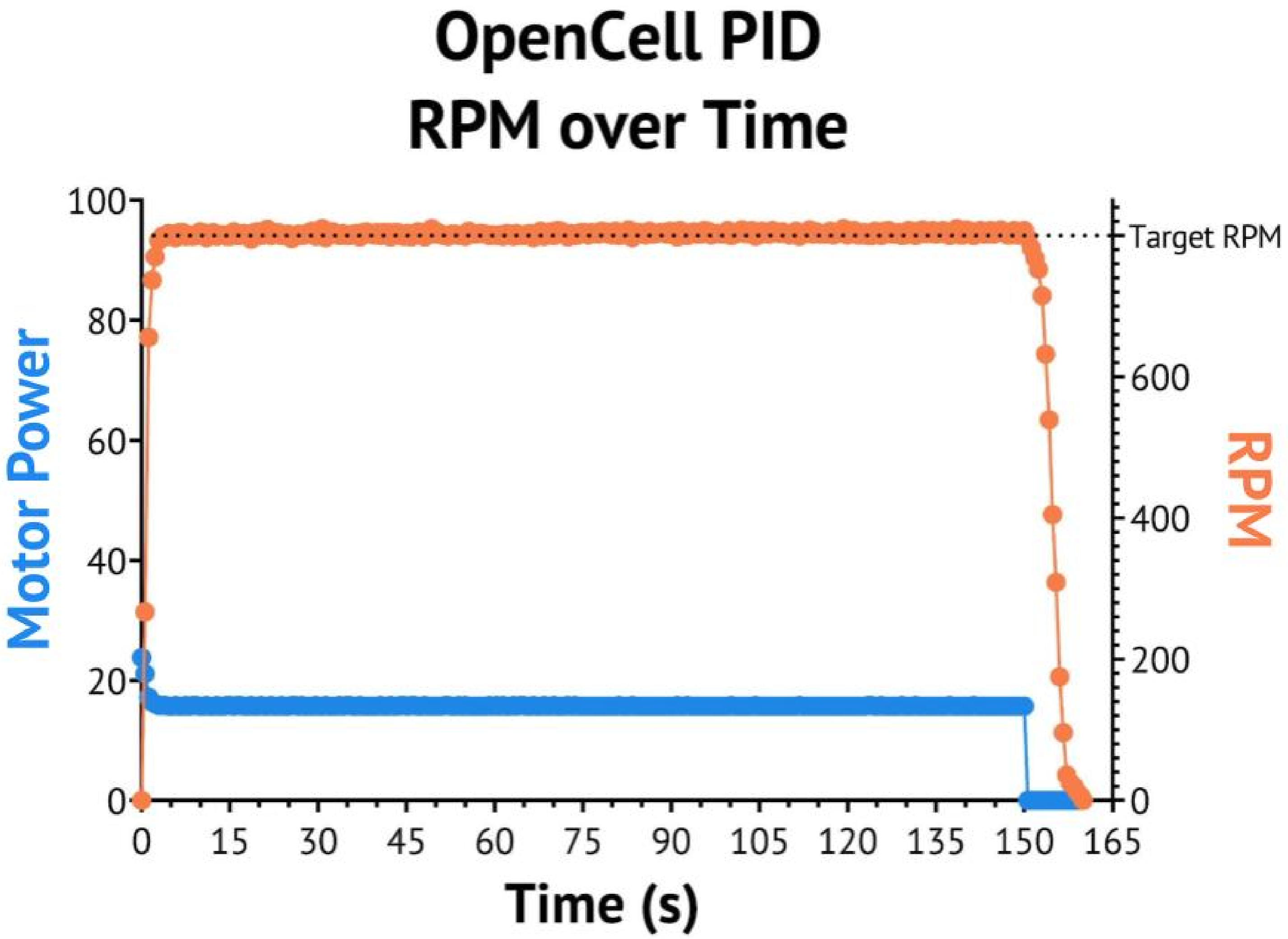

Supplement: S1 File — Diagram of all electronic components used in OpenCell, attachment speed over time using PID controller. (PDF) [file pone.0298857.s006.pdf]
