# Supplementary material for: OpenCell: A low-cost, open-source, 3-in-1 device for DNA extraction
Source: PLoS One. 2024 May 2;19(5):e0298857. doi: 10.1371/journal.pone.0298857 (PMC11065270; doi:10.1371/journal.pone.0298857)
Supplement: S1 Raw images — Uncropped, labeled, images of gels shown in Fig 4. (PDF) [file pone.0298857.s009.pdf]

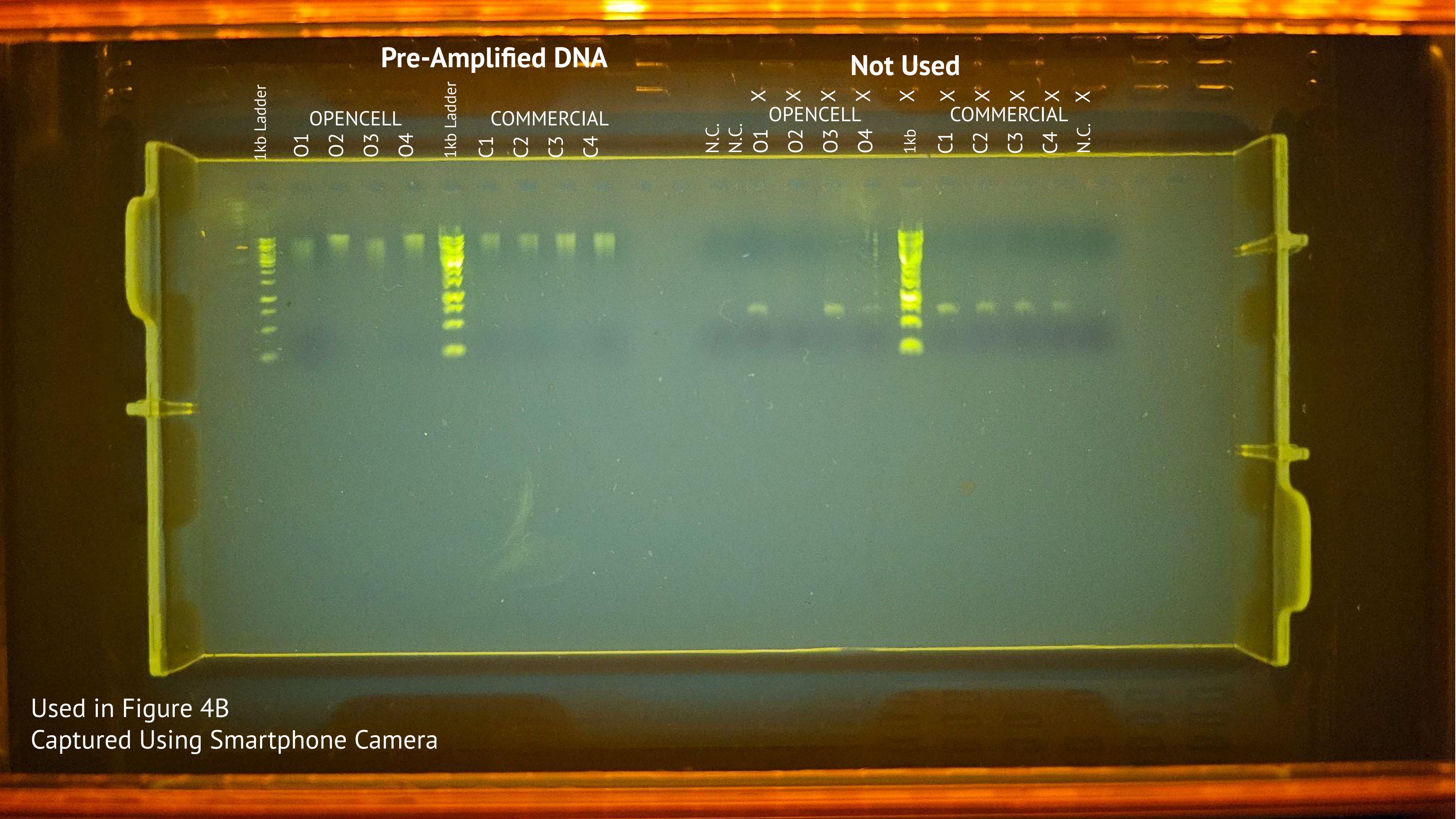

Used in Figure 4B
Captured Using Smartphone Camera

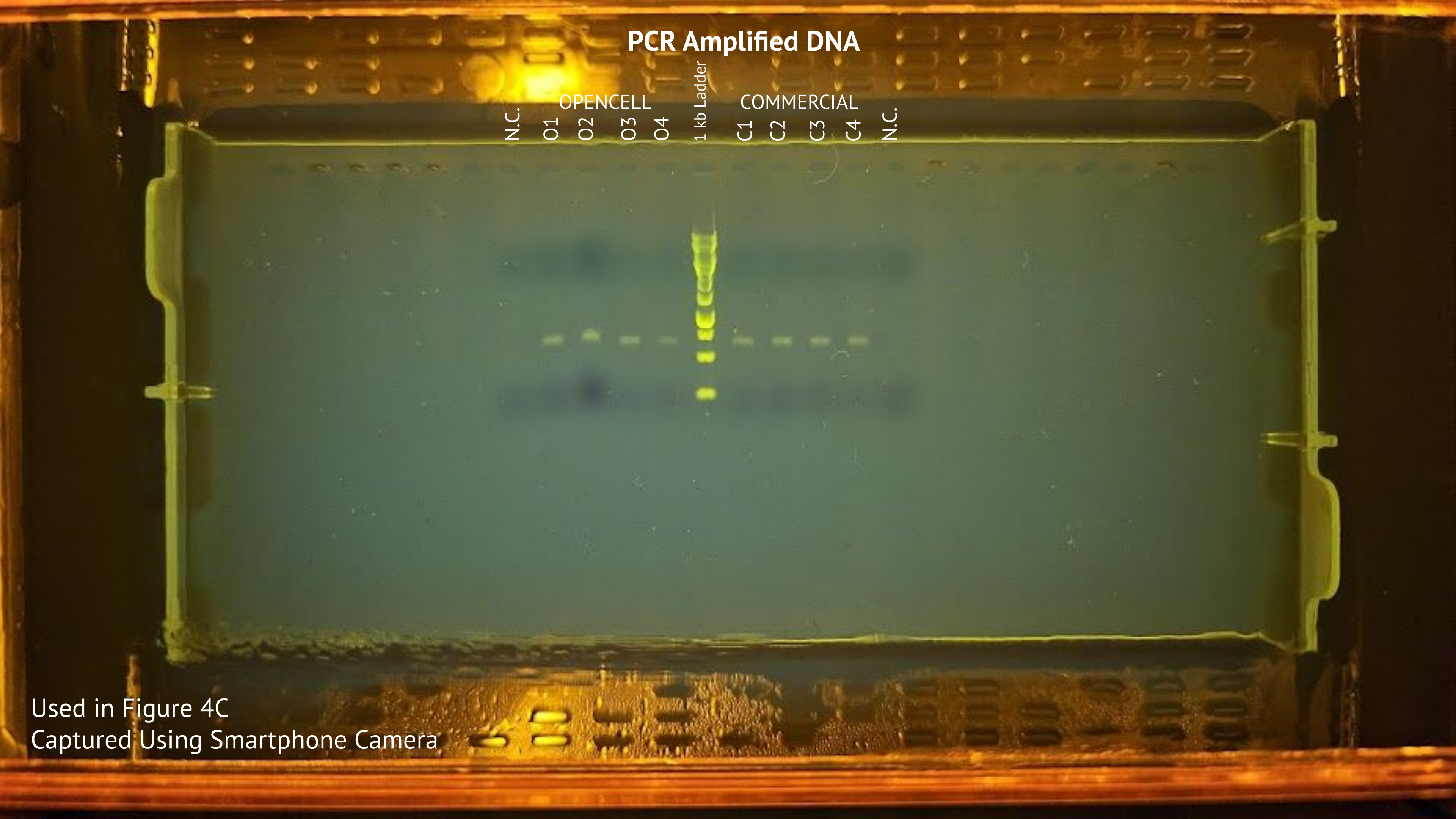

Used in Figure 4C
Captured Using Smartphone Camera
